# Assessing the Efficacy of Anterior Repositioning Splints in the Management of Temporomandibular Disc Displacement: A Systematic Review and Meta-Analysis

**DOI:** 10.7759/cureus.47689

**Published:** 2023-10-25

**Authors:** Shishir Dhar, Nitesh Kumar, Ashrafullah, Neha Dhaded, Prashant Hegde, Khushboo Chhabaria Peswani

**Affiliations:** 1 Oral and Maxillofacial Surgery, Ganesh Shankar Vidyarthi Memorial (GSVM) Medical College, Atal Bihari Vajpayee Medical University, Kanpur, IND; 2 Prosthodontics, Mansarovar Dental College, Hospital and Research Centre, Madhya Pradesh Medical Science University (MPMSU), Bhopal, IND; 3 Dentistry, Ganesh Shankar Vidyarthi Memorial (GSVM) Medical College, Atal Bihari Vajpayee Medical University, Kanpur, IND; 4 Endodontics, KLE Vishwanath Katti Institute of Dental Sciences and Hospital, Belgavi, IND; 5 Oral and Maxillofacial Surgery, Century Dental College, Kasargod, IND; 6 Oral and Maxillofacial Surgery, HT Hospital Cosmetic and Trauma Centre, Bhopal, IND

**Keywords:** tmj dysfunction, oral rehabilitation, jaw function, dental appliances, temporomandibular disorders, anterior repositioning splints, disc displacement, temporomandibular joint

## Abstract

Disc displacement (DD) occurs when the cushioning disc of the temporomandibular joint (TMJ), situated between the jawbone and the skull, is out of position. The condition can be of two kinds: disc displacement with reduction (ddwr) and disc displacement without reduction (ddwor). The present systematic review was undertaken to assess the efficacy of anterior repositioning splints (ARS) for ddwr and ddwor. Three online databases were searched for relevant studies using MeSH keywords and Boolean operators. Initial searches of the databases extracted 552 records. Twelve studies equally representing ARS with ddwr and ddwor were selected. No clear difference could be assessed regarding ARS usage in both conditions. The outcome assessed was the improvement in TMJ symptoms. The results suggested that both ddwr and ddwor were effective in improving temporomandibular joint (TMJ) symptoms in patients who received ARS therapy. Efficacy levels ranged from 71-83% and 50-95% for ARS in ddwr and ddwor, respectively; odds ratio (OR) values ranging from 0.30 and 0.36 were obtained for ARS in both therapies, respectively, indicating similar performance levels. Both ddwr and ddwor respond well to the use of ARS; however, more clinical trials are needed to ascertain and validate the role of ARS as a treatment modality in this regard.

## Introduction and background

Temporomandibular joint (TMJ) disorders refer to a range of conditions that affect the joints, muscles, and nerves of the jaw. The temporomandibular joint connects the jawbone to the skull and is responsible for the movement of the jaw when chewing, speaking, and yawning [1]. These can be caused by a variety of factors, such as injury to the jaw, bruxism, arthritis, stress, or misalignment of the teeth or jaw [2]. The symptoms of TMJ disorders manifest as pain or tenderness in the jaw, clicking sounds during movement of the jaw, difficulty chewing or speaking, tinnitus, vertigo and headaches [3].

There are several treatment modalities available for TMJ disorders, depending on the severity and underlying cause of the condition [4]. Pharmacological aid may include over-the-counter pain relievers that help relieve pain and reduce inflammation. Muscle relaxants and tricyclic antidepressants may also be prescribed to help manage symptoms [5]. A dental splint or mouthguard can help alleviate symptoms by protecting against clenching or grinding of the teeth. Dental treatments such as bite adjustments or orthodontic treatments may also be recommended to help realign the teeth and jaw. Exercises to strengthen the jaw muscles and improve the range of motion can help alleviate symptoms. Massage therapy, stretching, and injection of botox to the massetric muscle may also be recommended [6]. In severe cases, surgery may be necessary to repair or replace the temporomandibular joint. This may involve arthroscopy, a minimally invasive procedure, or open-joint surgery [7].

Another treatment modality is the utilisation of ARS, a type of dental splint that is sometimes used to treat TMJ disorders [8]. They are sometimes called "anterior bite planes" or "anterior deprogrammers." An ARS is a custom-made dental appliance to fit over the maxillary or mandibular teeth, and it is designed to move the lower jaw forward and away from the temporomandibular joint [9]. By doing so, an ARS can help reduce pressure on the joint, alleviate pain and discomfort, and improve jaw function. An ARS is typically made from acrylic or other durable materials and is worn for a certain period each day, usually several weeks or months. During this time, the patient may be advised to avoid hard or chewy foods and to perform certain exercises or stretches to help improve jaw function [10].

Specifically, an ARS is designed to alleviate pain and discomfort caused by the displacement or damage of the disc that cushions the joint. This can occur as a result of a jaw injury, clenching or grinding of the teeth, misalignment of the teeth or jaw, or other factors. It is intended to reposition the jaw in a way that reduces pressure on the temporomandibular joint, allowing it to heal and function properly [11]. It can also help alleviate related symptoms such as headaches, neck pain, and ear pain. In addition to TMJ disorders, an ARS may also be used in certain cases of bruxism or malocclusion [12].

Disc displacement (DD) refers to a condition in which the cushioning disc located between the jawbone and the skull in the TMJ is not correctly aligned [13]. This can occur for various reasons, such as trauma or injury to the joint, degenerative joint disease, or long-term grinding or clenching of teeth. There are two types of DD: ddwr and ddwor. In ddwr, the disc moves out of its normal position when the jaw is opened but then returns to its normal position when closed [14]. This type of disc displacement is often accompanied by a clicking or popping sound when the jaw is moved, but it may not cause any pain or discomfort. In ddwor, the disc remains out of position when the jaw is opened, and it does not return to its normal position when the jaw is closed. This type of DD can be associated with pain, discomfort, and restricted jaw movement [15]. Treatment options may include medication, physical therapy, dental appliances like anterior repositioning splints, or, in some cases, surgery [15].

In both cases, an ARS can be used to help alleviate symptoms and promote healing of the TMJ. For ddwr, the ARS is designed to reposition the jaw to reduce pressure on the joint and helps the disc return to its normal position. For ddwor, the ARS is designed to reposition the jaw to minimise stress on the joint and prevent further damage to the disc [16-17]. There are many studies on the use of ARS for TMJ disorders; still, these studies have produced mixed results, and the quality of the evidence may be limited due to small sample sizes or variations in study design [16-18]. Hence, this review was undertaken to synthesise evidence on the effectiveness of anterior repositioning splints for disc displacement conditions.

## Review

Material and methods

Review Framework and Registration

The Preferred Reporting Items for Systematic reviews and Meta-Analyses (PRISMA) protocol [19] was followed for the purpose of guiding the search strategy and its implementation. The PICO strategy employed for the review was- Population: patients diagnosed with temporomandibular joint disc displacement; Intervention: it included the application of anterior repositioning splints (ARS) therapy; Comparison: no treatment, other types of TMJ treatment, or a placebo intervention; and Outcome: Improvement in TMJ symptoms, including pain, clicking, jaw function, quality of life, and changes in disc position as measured by various imaging and diagnostic tests.

Using this PICO strategy, the systematic review and meta-analysis aimed to answer questions such as: "What is the overall effectiveness of anterior repositioning splints for treating TMJ disorders caused by disc displacement with and without reduction?" and "What are the potential benefits and harms of this treatment approach compared to other interventions or no treatment?"

Search Protocol Across Databases

The databases of PubMed, Web of Sciences, and Scopus were scoured for the identification and selection of relevant studies, the strategy of which is as follows. PubMed: ("temporomandibular joint disorders[Mesh]) OR ("temporomandibular joint disc displacement" [Me AND ("splints[Mesh] OR "orthotic devices" AND ("repositioning[Subheading] OR ading] OR "anterior" [ NOT ("surgery[Mesh] OR "drug therapy[Mesh] OR "physical therapy].

Web of Science: TS=("anterior repositioning splints" OR "anterior repositioning appliances" OR "anterior repositioning devices" OR "anterior repositioning orthotics") AND TS=("temporomandibular joint disorders" OR "temporomandibular joint disc displacement") NOT TS=("surgery" OR "drug therapy" OR "physical therapy"). 

Scopus: TITLE-ABS-KEY (anterior repositioning splints" OR "anterior repositioning appliances" OR "anterior repositioning devices" OR "anterior repositioning orthotics") AND TITLE-ABS-KEY("temporomandibular joint disorders" OR "temporomandibular joint disc displacement") NOT TITLE-ABS-KEY (surgery" OR "drug therapy" OR "physical therapy")

In all three databases, the search terms were combined using boolean operators (AND, OR, NOT) to refine the search and identify relevant studies. MeSH keywords were used in PubMed, while title/abstract keywords were used in Web of Science and Scopus. No time frame limitation was applied to the search. 

Eligibility Criteria

Studies that included adult patients with clinically diagnosed TMJ disorders and used ARS as the primary treatment modality or as an adjunct to other treatments were included, with no restriction on timeline. Case reports or case series, reviews, commentaries, and editorials were excluded. Studies that focused on other TMJ treatment modalities, such as pharmacotherapy, physical therapy, or surgical intervention, were excluded. Studies that did not report relevant outcomes such as pain, jaw function, or disc position were also excluded.

Data Extraction

The search was conducted by two independent reviewers who screened the titles and abstracts of identified studies for inclusion, followed by a full-text review of potentially eligible studies. Discrepancies between reviewers were resolved through discussion or a third reviewer. Data were extracted from the included studies by the same reviewers using a standardized form. Variables extracted from each study were study ID, publication year, type of disc displacement, sample size, age range, gender ratio, diagnostic technique, follow-up, and inferences.

Risk of Bias Assessment

The Risk of Bias-2 (ROB-2) [20] was used to assess the methodological quality of the studies included. The same reviewers assessed each included study using the tool. The tool assessed the risk of bias across six principal domains, and for each domain, the reviewers assessed the risk of bias as low, high, or unclear.

Protocol for Meta-Analysis

Review Manager 5.4 (RevMan Web, The Cochrane Collaboration, UK) was used for statistical analysis. Pooled results across studies using fixed-effects models in terms of the OR of ddwr and ddwor at a 95% confidence interval (CI) were tabulated. Forest plots were generated using the software to visualise the results of the meta-analyses and identify any heterogeneity or inconsistency across studies.

Results

**Figure 1:**
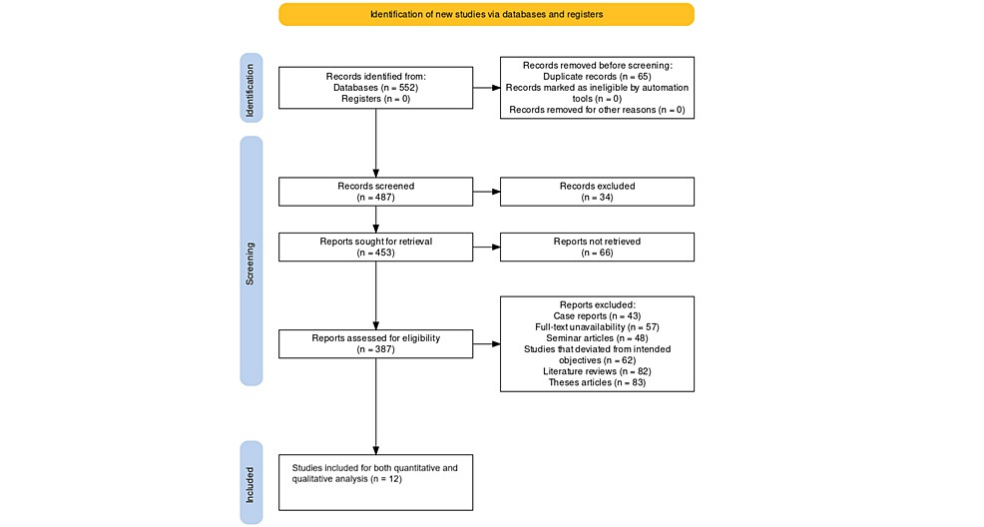
Framework representing the study selection protocol using PRISMA PRISMA: Preferred Reporting Items for Systematic Reviews and Meta-Analyses

As shown in Figure 1, after searching the online databases, initially 552 articles were retrieved. After the application of the different selection criteria that were devised for this review, we were ultimately left with 12 studies [21-32].

Table 1 shows the various characteristics of the studies eligible for review.

**Table 1:** Variables analysed in the included papers

Author	Year	With or without reduction	Sample strength (n)	Age range (in years)	Male: female ratio
Abdel et al. [21]	2021	With reduction	100	21-44	36:64
Di Paolo et al. [22]	2020	Without reduction	140	25-60	19:121
Diracoglu et al. [23]	2009	Without reduction	110	17-61	1:10
Huang et al. [24]	2011	With reduction	59	5-76	15:44
Kurt et al. [25]	2011	With reduction	35	26.8 (mean)	17:18
Lei et al. [26]	2020	Without reduction	20	12-30	1:19
Liu et al. [27]	2017	With reduction	37	18.8 (mean)	7:30
Ma et al. [28]	2019	With reduction	72	10-20	17:55
Muhtarogullari et al. [29]	2014	Without reduction	23	24-48	3:20
Pihut et al. [30]	2018	With reduction	112	24-45	29:83
Pihut et al. (2013) [31]	2013	Without reduction	32	19-38	Both genders included but ratio undefined
Schmitter et al. [32]	2005	Without reduction	74	18-72	9:65

Tables 2-3 showed the impact of ARS in ddwr and ddwor cases, respectively, alongside other information pertaining to the protocol employed and the follow-up periods in them.

**Table 2:** Details pertaining to studies where ddwr was observed ddwr: Disc displacement with reduction

Author	Protocol employed	Diagnostic aid used	Issues associated with ddwr	Overall impact of ARS and percentage of success	Follow-up period
Abdel et al. [21]	Comparative study	MRI	Clicking sound at mouth opening, pain during mastication	Positive (Percentage undefined)	Six months
Huang et al. [24]	Observational study	Undefined	Clicking sound at mouth opening, pain during mastication	Positive (71.2%)	12 months
Kurt et al. [25]	Comparative study	MRI	Clicking sound at mouth opening	Positive (Percentage undefined)	Six months
Liu et al. [27]	Observational study	MRI	Clicking sound at mouth opening, pain during mastication	Positive (83.78%)	Unavailable
Ma et al. [28]	Observational study	MRI	Clicking sound at mouth opening, pain during mastication	Positive (75.82%)	12 months
Pihut et al. [30]	Prospective study	MRI	Pain during mastication, improper contracting of masticatory muscles	Positive (Percentage undefined)	Four months

**Table 3:** Details pertaining to studies where ddwor was observed ddwor: Disc displacement without reduction

Author	Protocol employed	Diagnostic aid used	Issues associated with ddwor	Overall impact of ARS and percentage of success	Follow-up period
Di Paolo et al. [22]	Retrospective study	MRI	TMJ pain, clicking sound at mouth opening, pain during mastication, headache	Positive (70%)	12 months
Diracoglu et al. [23]	Prospective study	MRI	Clicking sound at mouth opening, pain during mastication	Positive (Percentage undefined)	Six months
Lei et al. [26]	Prospective study	MRI	Clicking sound at mouth opening	Positive (95.2%)	12 months
Muhtarogullari et al. [29]	Retrospective study	MRI	Clicking sound at mouth opening, pain during mastication	Positive (Percentage undefined)	24 weeks
Pihut et al. (2013) [31]	Retrospective study	Undefined	Clicking sound at mouth opening, pain during mastication	Positive (71.8%)	Four months
Schmitter et al. [32]	Comparative study	MRI	TMJ pain, clicking sound at mouth opening, pain during mastication	Positive (50%)	Six months

**Figure 2:**
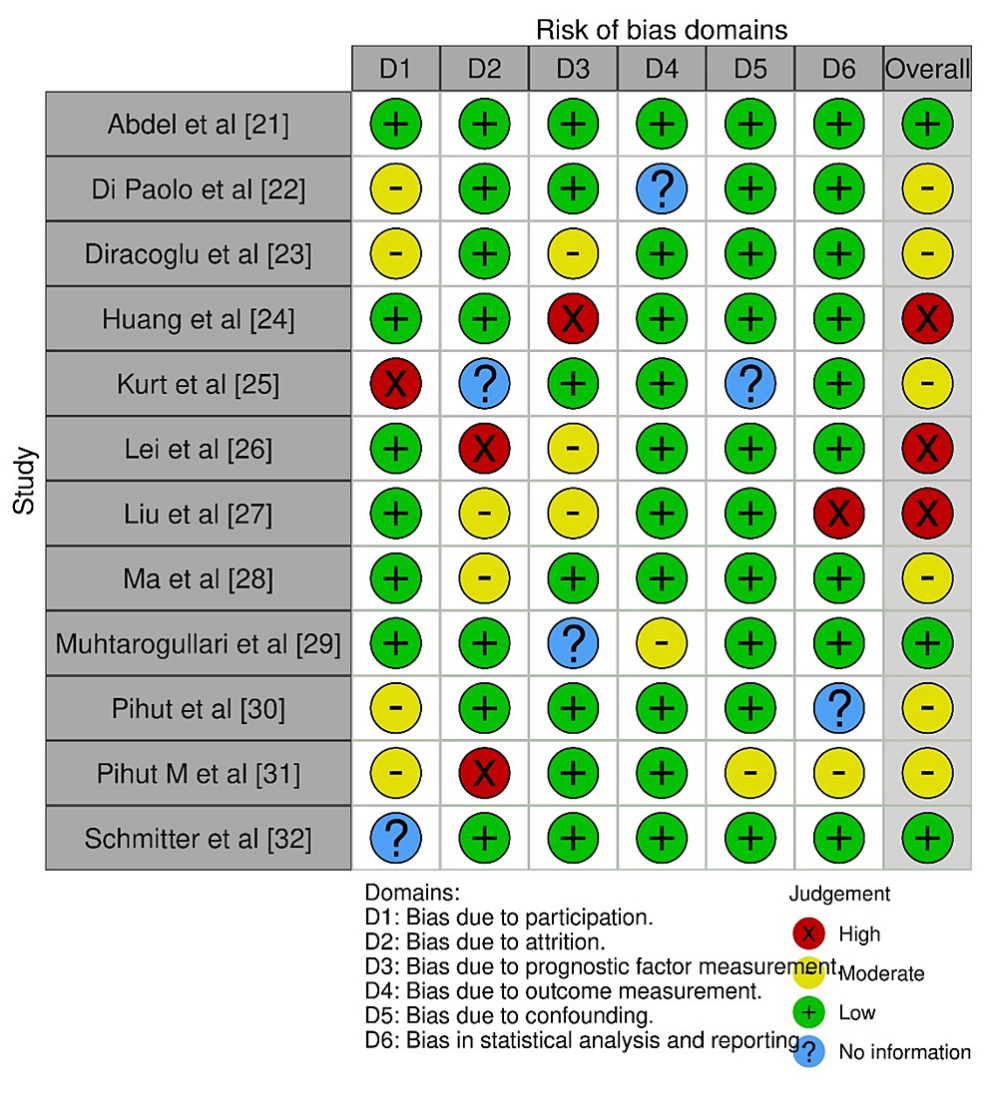
Bias assessment of the included studies Pihut M et al [31] = Pihut et al. (2013) [31]

Out of these 12 studies, six represented the cases where individuals were administered ARS for ddwr [21,24-25,27-28,30], whereas the other six cases were representative of the usage of ARS in cases of ddwor [22-23,26,29,31-32]. Three studies were retrospective in design [22,29,31], with three others being comparative trials [21,25,32] between ARS and other modalities. Three were observational [24,27-28], and the remaining three were prospective in protocol [23,26,30]. The age range across the studies was from five years to 72 years old. In all the studies [21-30,32], except for the study of Pihut et al. (2013) [31], a definite female predilection was observed. A positive response to the usage of ARS was seen in all the studies, irrespective of whether they were cases of ddwr or ddwor, with varying levels of percentage (all being significant) [21-32]. The methodological assessment of the quality of the included studies showed moderate to good quality studies, as shown in Figure 2.

**Figure 3:**
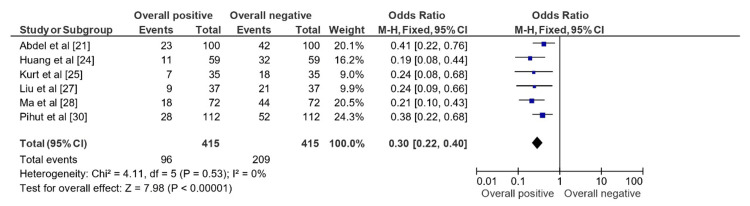
OR representation on forest plot displaying the impact of ARS usage in studies of cases representing ddwr OR: Odds ratio; ARS: Anterior repositioning splints; ddwr: Disc displacement with reduction

The OR of 0.30 (0.22, 0.40) (Figure 3) represents the summary effect estimate of the impact of ARS usage in studies of cases representing ddwr. The forest plot displaying the overall positive vs. overall negative impact of ARS usage indicates that the overall effect is statistically significant, as evidenced by the test for overall effect with Z = 7.98 (p < 0.00001). This means that the observed odds ratio of 0.30 is unlikely to have occurred by chance, and we can conclude that there is a true effect of ARS usage in studies of cases representing ddwr. The heterogeneity test indicates that there is no significant heterogeneity among the studies. This suggests that the studies are relatively consistent in their estimates of the effect of ARS usage and that the summary odds ratio is a reliable estimate of the true effect size. Overall, this indicates that ARS usage has a significant overall positive impact in studies of cases representing ddwr, with an OR of 0.30 (0.22, 0.40).

**Figure 4:**
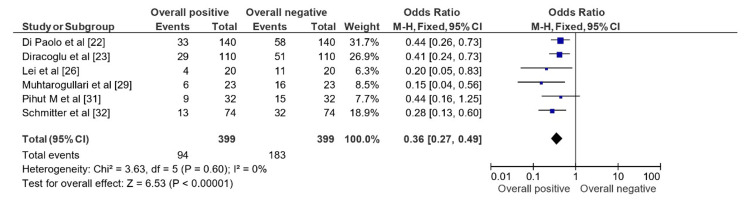
OR representation on forest plot displaying the impact of ARS usage in studies of cases representing ddwor OR: Odds ratio; ARS: Anterior repositioning splints Pihut M et al [31] = Pihut et al. (2013) [31]

Figure 4 shows an OR of 0.36 (0.27, 0.49) representing the summary effect estimate of the impact of ARS usage in studies of cases representing ddwor. The forest plot displaying the overall positive vs. overall negative impact of ARS usage indicates that the overall effect is statistically significant, as evidenced by the test for overall effect with Z = 6.53 (p < 0.00001). This means that the observed odds ratio of 0.36 is unlikely to have occurred by chance, and we can conclude that there is a true effect of ARS usage in studies of cases representing ddwor. No significant heterogeneity among the studies was reported, suggesting consistency in the effect estimate of ARS usage. The therapy demonstrated a significant overall positive impact in studies of cases representing ddwor, with an OR of 0.36 (0.27, 0.49).

Overall, the synthesised evidence suggests that ARS was effective in reducing TMD symptoms in both conditions of ddwr and ddwor.

Discussion

The review analysed a range of different study designs, encompassing both retrospective and prospective studies, as well as observational and comparative trials. The findings from this review suggest that ARS usage has a significant overall positive impact on both types of conditions, with summary effect estimates of 0.30 and 0.36 for ddwr and ddwor, respectively. One potential gap in the literature that this study addresses is the lack of systematic reviews and meta-analyses that have examined the impact of ARS usage in the treatment of ddwr and ddwor. By synthesising the available evidence from multiple studies, this review provides a more comprehensive understanding of the potential benefits of ARS usage in these conditions. The findings of this review have important implications for clinical practice. If confirmed by further research, these results suggest that ARS usage may be an effective treatment option for individuals with ddwr and ddwor. This could potentially improve outcomes and quality of life for individuals with these conditions. Additionally, these findings could help inform treatment decisions for clinicians working with individuals with these conditions.

Protruding the mandible is the beginning point of ARS therapy, and as a result, the procedure alters the interaction between the articular disc and condyle [25]. In the early stages of application, protruding the mandible causes the condyle to shift forward and "catch" the displaced disc [28]. This corrects the connection between the disc and the condyle and makes it easier for the tissue to adapt or heal. The disc-condyle complex is put back in its proper position as the mandible returns to its initial position after therapy is finished by gradually grinding the splint [23].

The concept of restoration, according to some medical professionals, is to move the condyle inferoanteriorly and let the disc reposition itself. According to a study that used MRI to study the discs of patients with ddwr, 41.9% of the discs that had been recovered at the edge-to-edge position were displaced once more during the process of backing to the least protrusive position [33]. The forward and downward condyle movements that fix the disc in a favourable position and keep it from shifting again during the mouth-closing process provided strong support for the hypothesis of another study, according to this finding [33]. A healthy disc-condyle connection can lessen the disc's collision, rebound, and release, which can stop chronic disc injury from occurring during abnormal movements [34]. Stress distribution also varies as internal structure changes. According to some studies, reducing unfavourable loading was a key factor in symptom relief [27,35]. Elastic tissues and articular cartilage may deteriorate as a consequence of pressure that results from stress that is concentrated in some areas [31].

Hard tissues are impacted by changes in stress distribution and undergo an adaptive shift as a result. Hollender and Lindahl [36] made the initial discovery of the "double contour" pictures while remodelling condylar fractures. The images visible in X-ray, CT, and MRI scans are typically recognised as significant indicators of development and remodelling. Another study found no significant difference in the incidence rate between teenage and adult groups and also found that patients with DD who received ARS therapy showed "double contour" images, which only appeared in the joints with improved internal structure [37].

The articular disc can become damaged by concentrated tension. As per the study of Machon et al. [38], anterior DD frequently results in distal puncture. The anterior displacement of the disc increased the maximum compressive stress to 14.6 times higher than its average score, with the maximum tensile stress reaching 1.43 MPa at the intermedial zone, which is in close proximity to tensile stress failure [39]. Tanaka et al. [40] further reinforced this observation. Anterior DD was associated with a five-fold rise in the shear stresses in the bilaminar zone. The bilaminar zone is an area of loose connective tissues, which is heavily vascularized and innervated and is referred to as the posterior attachment [36,40]. The only structure capable of retracting the disc posteriorly on the condyle is the superior retrodiscal lamina in the bilaminar zone [41]. Abnormal stress in this region may not only cause pain but may also decrease the elasticity of this region, leading to laceration or perforation. These findings suggest that compressing the disc may help decrease the concentration of local stress, thereby avoiding disc damage and relieving pain [42]. Experimental displacement using two different methods is required to determine how much ARS can alter the stress distribution. First off, after the splint is removed, an adaptive shift in the condyle encourages disc stabilisation and enhances the disc-condyle relationship. Second, among children and adolescents with TMD, ddwor is significantly associated with TMJ disorders [43]. TMD conditions can prevent the condyle from growing normally and result in malformation [44], but they do not have a reliable cure. According to Lei et al.'s research [45], ARS can help juvenile patients with early-stage TMJ degenerative joint disease (DJD) repair and regenerate their condyles and avert a poor prognosis for DD. These conclusions were supported by laboratory tests. Mandible protrusion has been shown by a couple of papers [46-47] to raise growth factor, hasten mesenchymal cell differentiation into chondrocytes, and increase chondrogenesis and osteogenesis in mandibular condyles. According to another study [48], the mandible protrusion may encourage cartilage development modification by activating genetic signalling pathways in the TMJ.

Study limitations

A limitation of this review was the small number of studies included. Although the review analysed a range of different study designs, only 12 studies met the inclusion criteria. This relatively small sample size may limit the generalizability of the findings, and further research is needed to confirm these results. Another limitation is the potential for publication bias. As with any meta-analysis, there is a risk that studies with negative findings may be underrepresented in the analysis due to publication bias. This could potentially overestimate the impact of ARS usage in the treatment of ddwr and ddwor. Moreover, the study did not examine potential adverse effects or long-term outcomes associated with ARS usage. This information could be important for clinicians considering ARS as a treatment option for individuals with ddwr and ddwor

## Conclusions

In conclusion, this review analysed a total of 12 studies that investigated the impact of ARS usage in cases of ddwr and ddwor. Overall, a positive response to the usage of ARS was seen in all studies, with varying levels of percentage, and the statistical analysis indicates that ARS usage has a significant overall positive impact in studies of cases representing both ddwr and ddwor. The summary effect estimates for the impact of ARS usage in studies of cases representing ddwr and ddwor were 0.30 and 0.36, respectively, and both were statistically significant. The studies were relatively consistent in their estimates of the effect of ARS usage, and the summary odds ratios are likely to be reliable estimates of the true effect sizes. Therefore, these findings suggest that ARS usage can be a valuable treatment option for both ddwr and ddwor.

## References

[1] Qamar Z, Alghamdi AM, Haydarah NK (2023). Impact of temporomandibular disorders on oral health-related quality of life: a systematic review and meta-analysis. J Oral Rehabil.

[2] Laskin D (1994). Etiology and pathogenesis of internal derangement of the temporomandibular joint. Oral Maxillofac Surg Clin North Am.

[3] Minervini G, Franco R, Marrapodi MM, Fiorillo L, Cervino G, Cicciù M (2023). Economic inequalities and temporomandibular disorders: a systematic review with meta-analysis. J Oral Rehabil.

[4] Stegenga B (2001). Osteoarthritis of the temporomandibular joint organ and its relationship to disc displacement. J Orofac Pain.

[5] Dolwick MF, Katzberg RW, Helms CA, Bales DJ (1979). Arthrotomographic evaluation of the temporomandibular joint. J Oral Surg.

[6] Katzberg RW, Dolwick MF, Helms CA, Hopens T, Bales DJ, Coggs GC (1980). Arthrotomography of the temporomandibular joint. AJR Am J Roentgenol.

[7] Westesson PL (1983). Double-contrast arthrotomography of the temporomandibular joint: introduction of an arthrographic technique for visualization of the disc and articular surfaces. J Oral Maxillofac Surg.

[8] Bronstein SL, Tomasetti BJ, Ryan DE (1981). Internal derangements of the temporomandibular joint: correlation of arthrography with surgical findings. J Oral Surg.

[9] Okeson JP (2003). Occlusal appliance therapy. Management of Temporomandibular Disorders and Occlusion (5th Edition).

[10] Dworkin SF, LeResche L (1992). Research diagnostic criteria for temporomandibular disorders: review, criteria, examinations and specifications, critique. J Craniomandib Disord.

[11] Mintz SS (1993). Craniomandibular dysfunction in children and adolescents: a review. Cranio.

[12] Okeson JP (1996). Orofacial pain: guidelines for assessment, diagnosis, and management. USA: Quintessence Publishing Co Inc.

[13] Forssell H, Kalso E (2004). Application of principles of evidence-based medicine to occlusal treatment for temporomandibular disorders: are there lessons to be learned?. J Orofac Pain.

[14] de Leeuw R, Klasser GD (2013). Diagnosis and Management of TMDs. Orofacial Pain: Guidelines for Assessment, Diagnosis, and Management.

[15] Chen HM, Liu MQ, Yap AU, Fu KY (2017). Physiological effects of anterior repositioning splint on temporomandibular joint disc displacement: a quantitative analysis. J Oral Rehabil.

[16] Shedden Mora MC, Weber D, Neff A, Rief W (2013). Biofeedback-based cognitive-behavioral treatment compared with occlusal splint for temporomandibular disorder: a randomized controlled trial. Clin J Pain.

[17] Guo YN, Cui SJ, Zhou YH, Wang XD (2021). An overview of anterior repositioning splint therapy for disc displacement-related temporomandibular disorders. Curr Med Sci.

[18] Miernik M, Więckiewicz W (2015). The basic conservative treatment of temporomandibular joint anterior disc displacement without reduction--review. Adv Clin Exp Med.

[19] Liberati A, Altman DG, Tetzlaff J (2009). The PRISMA statement for reporting systematic reviews and meta-analyses of studies that evaluate health care interventions: explanation and elaboration. Ann Intern Med.

[20] McGuinness LA, Higgins JP (2021). Risk-of-bias VISualization (robvis): An R package and Shiny web app for visualizing risk-of-bias assessments. Res Synth Methods.

[21] Abdel-Gawwad EA, Abdullah AA, Farhat MY, Helal MA (2021). Effect of using Photobiomodulation, stabilization, and anterior repositioning splints on the pain level of subjects with temporomandibular joint disc displacement with reduction. Brazilian Dental Science.

[22] Di Paolo C, Falisi G, Panti F, Di Giacomo P, Rampello A (2020). "RA.DI.CA." Splint for the Management of the Mandibular Functional Limitation: A Retrospective Study on Patients with Anterior Disc Displacement without Reduction. Int J Environ Res Public Health.

[23] Diraçoğlu D, Saral IB, Keklik B (2009). Arthrocentesis versus nonsurgical methods in the treatment of temporomandibular disc displacement without reduction. Oral Surg Oral Med Oral Pathol Oral Radiol Endod.

[24] Huang IY, Wu JH, Kao YH, Chen CM, Chen CM, Yang YH (2011). Splint therapy for disc displacement with reduction of the temporomandibular joint. part I: modified mandibular splint therapy. Kaohsiung J Med Sci.

[25] Kurt H, Mumcu E, Sulun T, Diracoglu D, Unalan F, Aksoy C, Tuncer N Comparison of Effectiveness of Stabilization Splint, Anterior Repositioning Splint and Behavioral Therapy in Treatment of Disc Displacement with Reduction. Turkiye Fiziksel Tip ve Rehabilitasyon Dergisi.

[26] Lei J, Yap AU, Li Y, Liu MQ, Fu KY (2020). Clinical protocol for managing acute disc displacement without reduction: a magnetic resonance imaging evaluation. Int J Oral Maxillofac Surg.

[27] Liu MQ, Lei J, Han JH, Yap AU, Fu KY (2017). Metrical analysis of disc-condyle relation with different splint treatment positions in patients with TMJ disc displacement. J Appl Oral Sci.

[28] Ma Z, Xie Q, Yang C, Zhang S, Shen Y, Abdelrehem A (2019). Can anterior repositioning splint effectively treat temporomandibular joint disc displacement?. Sci Rep.

[29] Muhtarogullari M, Avci M, Yuzugullu B (2014). Efficiency of pivot splints as jaw exercise apparatus in combination with stabilization splints in anterior disc displacement without reduction: a retrospective study. Head Face Med.

[30] Pihut M, Gorecka M, Ceranowicz P, Wieckiewicz M (2018). The efficiency of anterior repositioning splints in the management of pain related to temporomandibular joint disc displacement with reduction. Pain Res Manag.

[31] Pihut M, Wiśniewska G, Majewski S Active repositioning of temporomandibular disc displacement without reduction. Journal of Stomatology.

[32] Schmitter M, Zahran M, Duc JM, Henschel V, Rammelsberg P (2005). Conservative therapy in patients with anterior disc displacement without reduction using 2 common splints: a randomized clinical trial. J Oral Maxillofac Surg.

[33] William K, Solberg GTC (1980). Temporomandibular joint problems: Biological diagnosis and treatment.

[34] Chen HM, Fu KY, Li YW, Zhang ZK (2009). Positional changes of temporomandibular joint disk and condyle with insertion of anterior repositioning splint (Article in Chinese). Hua Xi Kou Qiang Yi Xue Za Zhi.

[35] Okeson JP (2019). Management of temporomandibular disorders and occlusion, 7th Edition. https://www.academia.edu/44984931/Jeffrey_Okeson_Management_of_Temporomandibular_Disorders_and_Occlusion_Mosby_Elsevier_2019_SEMINARIO_DTM.

[36] Lindahl L, Hollender L (1977). Condylar fractures of the mandible: II. A radiographic study of remodeling processes in the temporomandibular joint. International Journal of Oral Surgery.

[37] Pathria MN, Chung CB, Resnick DL (2016). Acute and stress-related injuries of bone and cartilage: pertinent anatomy, basic biomechanics, and imaging perspective. Radiology.

[38] Machon V, Levorova J, Hirjak D, Drahos M, Foltan R (2017). Temporomandibular joint disc perforation: a retrospective study. Int J Oral Maxillofac Surg.

[39] Liu Z, Qian Y, Zhang Y, Fan Y (2016). Effects of several temporomandibular disorders on the stress distributions of temporomandibular joint: a finite element analysis. Comput Methods Biomech Biomed Engin.

[40] Tanaka E, Rodrigo DP, Miyawaki Y, Lee K, Yamaguchi K, Tanne K (2000). Stress distribution in the temporomandibular joint affected by anterior disc displacement: a three-dimensional analytic approach with the finite-element method. J Oral Rehabil.

[41] Yano K, Nishikawa K, Sano T, Okano T (2009). Relationship between appearance of a double contour on the mandibular condyle and the change in articular disc position after splint therapy. Oral Surg Oral Med Oral Pathol Oral Radiol Endod.

[42] Liu MQ, Chen HM, Yap AU, Fu KY (2012). Condylar remodeling accompanying splint therapy: a cone-beam computerized tomography study of patients with temporomandibular joint disk displacement. Oral Surg Oral Med Oral Pathol Oral Radiol.

[43] Moncada G, Cortés D, Millas R, Marholz C (2014). Relationship between disk position and degenerative bone changes in temporomandibular joints of young subjects with TMD. An MRI study. J Clin Pediatr Dent.

[44] Hu YK, Yang C, Cai XY, Xie QY (2016). Does condylar height decrease more in temporomandibular joint nonreducing disc displacement than reducing disc displacement?: A magnetic resonance imaging retrospective study. Medicine (Baltimore).

[45] Lei J, Yap AU, Liu MQ, Fu KY (2019). Condylar repair and regeneration in adolescents/young adults with early-stage degenerative temporomandibular joint disease: a randomised controlled study. J Oral Rehabil.

[46] Rabie AB, She TT, Hägg U (2003). Functional appliance therapy accelerates and enhances condylar growth. Am J Orthod Dentofacial Orthop.

[47] Rabie AB, Xiong H, Hägg U (2004). Forward mandibular positioning enhances condylar adaptation in adult rats. Eur J Orthod.

[48] Sun L, Zhao J, Wang H, Pan Y, Wang L, Zhang WB (2017). Mechanical stress promotes matrix synthesis of mandibular condylar cartilage via the RKIP-ERK pathway. J Mol Histol.

